# Measuring the financial burden of acute cough in pre-school children: a cost of illness study

**DOI:** 10.1186/1471-2296-9-10

**Published:** 2008-01-31

**Authors:** Sandra Hollinghurst, Catherine Gorst, Tom Fahey, Alastair D Hay

**Affiliations:** 1 Academic Unit of Primary Health Care, University of Bristol, 25 Belgrave Road, Clifton, Bristol, UK; 2 School of Medical Education, University of Liverpool, Cedar House, Ashton Street, Liverpool, UK; 3 Department of General Practice and Family Medicine, Royal College of Surgeons in Ireland Medical School, 120 St Stephens Green, Dublin, Ireland

## Abstract

**Background:**

*Context: *Acute cough is a very common symptom presentation among children in primary care and is usually due to respiratory infection, yet its cost is unknown. An estimate of the cost to healthcare providers and parents would aid budgetary decision-making, and provide an insight into the need for interventions to reduce the burden. *Purpose: *To estimate the cost per child per episode, and the annual population cost in the UK, of acute cough in pre-school children presenting to primary care.

**Methods:**

*Design*: Incidence and prevalence-based cost-of-illness study from the perspectives of the UK NHS and of parents and caregivers. *Setting: *11 general practices in Bristol, UK. *Subjects: *121 children without known asthma aged 3 to 59 months presenting for the first time with an acute (≤ 28 days) cough.

**Results:**

Mean cost per episode to the NHS: £27.43 (95% CI: £24.38 – £30.49). Mean cost per episode to parents and carers: £14.77 (£4.90 – £24.65). Annual cost to the NHS in the UK: at least £31.5 m (95% CI: £28.0 m – £35.0 m).

**Conclusion:**

The cost burden on the healthcare provider of acute cough in pre-school children is substantial; the majority of this cost arises from consultations with general practitioners. Parents experience some personal cost through travel and expenditure on over-the-counter preparations, and may suffer significantly if loss of earnings is experienced. There is scope for evaluating interventions designed to reduce this burden.

## Background

Cough in children is the most commonly managed symptom in the NHS [1] and is usually associated with viral infection [2]. This is likely to generate a considerable cost burden as suggested by Ehlken et al [3], who estimated the mean cost of an episode of lower respiratory tract infection in children under the age of three treated as outpatients in Germany, Lambert et al [4], whose cohort study of children aged between 12 and 71 months estimated the cost of a community-managed episode of respiratory viral infection, and Fendrick [5] who conducted a study of viral respiratory tract infection in the general population of the USA. These studies vary in their scope and settings but there is agreement about the importance of the cost to both the healthcare provider and to parents. To our knowledge no research to date has attempted to estimate the scale of this burden in the UK. An estimate of these costs would indicate the relative importance of this issue to policy makers, clinicians, and parents. It would provide information to aid budgetary decision-making, and insight into the need for interventions to reduce the cost burden. The aim of this study was to estimate the annual cost to the National Health Service (NHS) in the UK, and the direct cost per episode to parents and carers, of acute cough in pre-school children presenting to primary care.

## Methods

This study was part of a larger cohort study designed to validate a clinical rule for predicting complications of acute cough in pre-school children [6]. Within this framework we carried out a cost-of-illness study on those children presenting for the first time for that episode of illness. This was conducted from the perspectives of the NHS and parents and caregivers. We estimated the cost to the NHS of an episode of illness and combined it with data from the literature to estimate the annual cost burden to the NHS in the UK. We also estimated the cost per episode incurred by parents and carers of children with acute cough. Participants included children without known asthma or other chronic disease aged 3 to 59 months who presented to 11 general practices in Bristol between September 2004 and May 2005, for the first time with an acute (≤ 28 days) cough.

### Identification of resource use and data collection

The provider perspective included all resources supplied by the NHS and used by participants during the period between the index consultation and cough resolution up to a maximum of four weeks following the initial consultation. These were identified as: practice based consultations with a doctor or nurse; telephone consultations; home visits; visits to a Walk-in Centre; contacts with NHS Direct; out-of-hours care including telephone consultation, face-to-face consultation and home visits; visits to A&E; out-patient appointments; in-patient hospital care; and prescribed medication.

From the perspective of the parents and caregivers, the relevant direct costs included: travel to health care facilities for visits associated with the child's cough; over-the counter medication purchased; extra care for dependents required because of the child's illness; and loss of earnings as a result of the child's illness.

Data on resource use were collected using a telephone questionnaire. A researcher contacted parents and carers weekly and asked about any contacts with a health care professional and any prescriptions received during the previous week. They were also asked about mode of travel and the cost of fares when visiting health care facilities, expenditure on over-the-counter preparations for cough, how much they had lost in terms of earnings foregone because of their child's illness, and any extra expenditure on child or other dependent care.

### Valuation of resources

All resources were valued in pounds sterling at 2006 prices. Unit costs and their source are given in Table 1. Resource use was valued using recognised sources of unit costs. Primary care contacts were valued using Curtis and Netten [7] and visits to the walk-in centre were valued using information from the national evaluation of first-wave walk-in centres [8]. Hospital-based care was valued using the NHS tariff [9], and for prescribed medication we used costs reported in the British National Formulary [10]. The AA schedule of motoring costs [11] was used to cost the use of cars for travel. Unit costs were adjusted, where necessary, using an appropriate inflation index [7]. No discounting was necessary as the time period involved was one month.

**Table 1:** Source and value of unit costs

	**£**	***Source***	***Details***
GP at surgery	21.00	Curtis and Netten 2006 [7]	Includes direct care staff costs but excludes cost of training
Nurse at surgery	8.00	Curtis and Netten 2006 [7]	
GP telephone consultation	23.00	Curtis and Netten 2006 [7]	Includes direct care staff costs but excludes cost of training
GP home visit	60.00	Curtis and Netten 2006 [7]	Includes direct care staff costs but excludes cost of training
Walk-in centre (WiC) with a nurse	28.91	WiC evaluation [8]	Mean of shop front & GP based, inflated to 2006
A & E visit	70.62	NHS Tariff [9]	A & E weighted average of referred/discharged using 2004 volume proportions
Out-patient appointment	19.85	NHS Tariff [9]	Band A x-ray cost in 2004 inflated
Prescribed medication		British National Formulary [10]	
Mileage	0.49	The AA [11]	Mean of mid-price car; 10,000 – 15,000 miles pa; petrol & diesel

### Estimating the annual cost to the NHS

The estimated cost per episode to the NHS from the cost-of-illness study was combined with estimates from the literature and routine sources to estimate the annual cost to the NHS of children presenting to primary care with a cough. The annual population cost was estimated as:

(cost per episode per child consulting) × (mean number of episodes per annum) × (population)

The mean number of episodes per annum is a combination of the number of (different) children consulting with a cough in any year and the mean number of episodes of illness per child per annum. There is evidence in the literature of the number of children consulting with one or more episodes of cough in any year [12], but we were unable to find evidence on the mean number of episodes of cough per child per annum. We therefore used a conservative estimate of one for our baseline analysis. The number of children in the UK under the age of five was taken from the Office of National Statistics [13]. All analyses were carried out using Microsoft Excel and Stata (version 9).

## Results

### Participant characteristics

Two hundred and fifty six eligible children were invited to take part in the cohort study to validate the prediction rule. Fifty-nine (23%) refused and 33 (13%) were unable to read or write English. Of the 164 recruited, 13 (8%) failed to complete or provide data and 30 (18%) were not consulting for the first time. Thus 121 children were suitable for inclusion in the cost-of-illness study. Recruitment was through general practices covering a broad socio-demographic spectrum. Compared with the general pre-school population our sample of children was a little younger (73% under the age of three compared with 61%) and contained more boys (56% vs. 51%). It was predominantly white (76%), though probably less than the general population (92% white in the UK at the 2001 census), with the remaining children split between a range of ethnic groups. Diagnostic labels assigned by the GPs (95%) or nurses (5%) seeing the children were: upper respiratory tract infection (64%), lower respiratory tract infection (9%), bronchiolitis (5%), bronchitis (4%), croup (3%), respiratory tract infection with bronchospasm (3%) and other (10%).

### Resource use

The mean resource use per child per episode, by item, is given in Table 2. One hundred and three (85%) children had only one primary care contact, thus the re-consultation rate was 15%. Nearly all (95%) of the primary care contacts were with a GP, the remainder with a nurse. One child visited A&E, and one child had an outpatient appointment for a chest x-ray. Prescriptions were issued to 58 (48%) children, with 36 (30%) receiving antibiotics. Parents of 35 (29%) children bought at least one over-the-counter preparation, with one parent purchasing four. Twenty-five (21%) parents reported losing income because of their child's illness and of these, two days off work was most common.

**Table 2:** Mean resource use per episode per participant

**Item of resource use**	***Mean (sd)***
**Primary Care**	
GP consultations	1.09 (0.48)
Nurse consultations	0.06 (0.23)
Telephone consultations	0.02 (0.13)
Home visits	0.01 (0.09)
Walk-in Centre visits	0.02 (0.13)
	
**Secondary Care**	
A & E visits	0.01 (0.09)
Out-patients appointments	0.01 (0.09)
	
**Prescriptions**	0.58 (0.69)
	
**Parental resource use**	
Primary care journeys	0.61 (0.62)
Secondary care journeys	0.02 (0.13)
Over-the-counter medication	0.43 (0.78)
Days off work	0.64 (1.71)

### Cost per episode

The mean cost per child per episode is given in Table 3. Including the initial consultation and during the period prior to cough resolution, the mean cost to the NHS per child was £27.43 (95% CI: £24.38, £30.49). Primary care accounted for 93% of this, with GP consultations costing £22.91 (£21.08, £24.73). Secondary care and prescribed medication costs were small. The mean cost per episode to parents and caregivers was £14.77 (£4.90, £24.65). Lost earnings accounting for the greatest part (85%) though there was considerable variation of experience among families. Mean expenditure per child on over-the-counter preparations was £1.32 (£0.89, £1.76), with the average purchase cost of a single preparation being £3.08 (£2.84, 3.32).

**Table 3:** Mean cost per episode per participant

**Item of resource use**	**Mean and (sd) cost (£)**
**Primary Care**	
GP consultations	22.91 (10.14)
Nurse consultations	0.46 (1.88)
Telephone consultations	0.38 (2.94)
Home visits	0.50 (5.45)
Walk-in Centre visits	0.48 (3.70)

**Total primary care**	**24.73 (12.75)**
	
**Secondary Care**	
A & E visits	0.58 (6.42)
Out-patients appointments	0.16 (1.80)

**Total secondary care**	**0.75 (6.65)**
	
**Prescribed medication**	**1.96 (3.54)**

**Total NHS costs**	**27.43 (16.99)**
	
**Parent and caregiver costs**	
Primary care travel	0.78 (1.21)
Secondary care travel	0.11 (0.90)
Over-the-counter medication	1.32 (2.43)
Lost earnings	12.56 (54.64)

**Total parent and care-giver costs**	**14.77 (54.84)**

### Annual cost to the NHS

We combined the cost per episode results with data on incidence and population. Evidence suggests that around 333 per thousand (i.e. a third) children under the age of five consult in primary care with cough at least once a year [12]. The population of under-fives in the UK in 2005 was 3.43 m [13]. Thus the annual cost to the NHS in 2006 was at least £31.5 m (95% CI: £28.0 m to £35.0 m).

### Sensitivity analysis

There are a number of reasons why our population cost estimate may be conservative and the effect of two of these is explored here. First, the cost per episode is based on the empirical work reported here. In our study the reconsultation rate was 15%. This is at the lower end of an observed range of reconsultation rates that include 17% [14] and 24% [15] in children with acute upper respiratory tract infection. Using a re-consultation rate of 20%, which may be more realistic, the population cost estimate rises to £32.7 m. Second, although we know that children experience between three and six respiratory tract infections per annum [2], we were unable to find evidence of the mean number of episodes of cough for which a child consults each year. We used an estimate of one but if 10% of children had consulted for two episodes of illness in a year, the annual cost estimate rises to £34.6 m.

## Discussion

The cost burden to the NHS of acute cough in pre-school children, at over £30 m, is substantial and the majority of this cost arises from consultations with general practitioners. This cost represents 1.3 million consultations each year in the UK. Parents experience some personal cost through travel and expenditure on over-the-counter preparations, and may suffer significantly if loss of earnings is experienced.

We have highlighted a number of reasons why our estimate may be conservative. A further factor is the limitation of our recruitment strategy. We were only able to recruit children who presented to primary care during normal office hours, thus children presenting initially to A&E or to out-of-hours primary care providers were not included. These children are likely to have more serious illness and may represent a greater cost burden. Also, we only included resource use from the time of the first GP consultation so any use of healthcare prior to that point will have been excluded. In addition, our cost estimate is limited by the poor data on indirect costs. Whilst we were able to estimate the direct cost to parents of time off work, the societal cost of that time is unknown. In total, the time off work due to childhood cough is substantial though this composed of a large number of short periods of absenteeism. The valuation of productivity loss is contentious and several methods have been proposed [16], the most appealing for pragmatic research being the friction cost method, a variation of the human capital approach, which includes only the resources required to replace the employee. Because of the disparate nature of the absenteeism here it is impossible to estimate the cost of lost productivity using the friction cost method without more detailed information on how the absenteeism was dealt with by employers. This conservative approach means that the true cost to society, including the cost of lost productivity, would also inflate the estimate. Despite this, our results are useful in indicating the cost burden of acute cough to the NHS and to parents and caregivers, and there is face validity to these estimates when compared with those of a recent study of the minimum cost of preschool asthma and wheeze [17]. Other studies of the cost of respiratory tract infections concur. Although they address different patient groups in different settings, the studies by Ehlken [3] and Lambert [4] also suggest our estimates may be conservative.

There is very little information available to compare the cost of cough with other reasons children consult in general practice. The most common symptoms are: stomach ache, fever, rash, and diarrhoea and vomiting [12]. Unpublished results of a current study indicate the cost to the NHS in the UK of fever in pre-school children is likely to be less than that of cough due to lower consultation rates per episode of illness and lower prevalence. Another common reason for children to use primary care is for routine immunisations. The cost of these is around £40 per child over the first five years of life, suggesting an annual cost to the NHS similar to that found here.

As recommended by the Medical Research Council [18], we chose a symptom (cough) rather than diagnosis or disease-based entry criterion for this study because of the inconsistency of diagnostic label use for respiratory tract infections in primary care [19]. Our study could therefore be regarded as a 'cost of symptom' study (rather than 'cost of illness'). Nevertheless, interpretation of the results is unaffected by this, as it is the symptom rather than the 'illness' that instigates the consultation and any intervention designed to reduce consultation rates would need to target the symptom (cough). Cost-of-illness studies can be viewed as being 'incidence based' or 'prevalence based' [20] and each has a different value to planners of health care services. Here we have performed both types of study by estimating the cost per episode of illness and the annual cost burden to the NHS. The prevalence approach indicates the overall scale of the cost burden, allows for comparison with other areas of health care, and can inform decisions as to whether cost savings are possible and/or desirable. Although as Fleurence and Torgerson [21] point out, one disadvantage of cost of illness studies is the limited extent to which they can inform the research agenda, this study shows that information provided by such a study is necessary if not sufficient to evaluate the need for future research.

The incidence approach is useful from an intervention perspective, indicating the scope for cost savings as a result of different treatment alternatives. There is evidence that delayed prescribing supported by patient information leaflets can affect healthcare-seeking behaviour [22], and information leaflets alone can affect reconsultation rates and patient behaviour [23,24]. Although no study has focussed on young children with cough there is an indication that such interventions could reduce antibiotic use and reconsultation rates considerably without affecting clinical outcomes [25]. In this study, if antibiotic use and reconsultations had been 50% lower, our estimate of cost per episode would be £2.04 lower and the annual cost to the NHS reduced by £2.3 m to £29.1 m. Therefore, an intervention costing less than £2.3 m, which reduces re-consultations by 50%, would be cost saving. Furthermore, delayed prescribing can change patient attitudes and lessen the need to consult in the future thus reducing costs to the NHS even further, though possibly at the expense of parents.

This study raises several questions that could be addressed by future research. Cost of illness studies address only the cost of a disease, (or as in this case, a symptom). They do not address outcomes and do not compare the cost and outcomes of alternative strategies, as an economic evaluation would. In order to assess the full significance of the results the costs need to be considered in the light of benefits arising from the expenditure. Most of the cost is general practitioner time so the benefits of a consultation must be identified. Two main outcomes are likely to be: reassurance for parents anxious to confirm that the illness is self-limiting; and the identification of children who are seriously ill, for example, have pneumonia, an infectious illness such as whooping cough, or have asthma. The value of these benefits is unknown and subjective.

Efficiency in delivering healthcare can be addressed by improving benefits or reducing the level of resource use or both. Exploiting the skill-mix within a practice could reduce resources used in delivering primary care to young children with cough. Practice nurses, trained to identify symptoms of serious illness, could be used to provide reassurance and deliver an educational intervention designed to inform parents about the likelihood of acute cough developing into a more serious condition and the symptoms that indicate deterioration. Although there is uncertainty about the overall relative cost-effectiveness of nurses and doctors in primary care [26], matching tasks to skills and experience may result in more efficient use of staff resources.

## Conclusion

This study has estimated the cost of one of the major reasons that children use primary care. The scale of this burden is large and indicates there is scope for evaluating the effectiveness and cost-effectives of interventions designed to reduce this burden.

## Competing interests

The author(s) declare that they have no competing interests.

## Authors' contributions

AH and TF had the idea for the study and SH designed it. CG recruited patients and collected the data. SH analysed the data and all authors contributed to the interpretation. SH wrote the first draft of the manuscript and all authors contributed to, read and approved the final manuscript.

## Pre-publication history

The pre-publication history for this paper can be accessed here:


